# Validating Automated Segmentation Tools in the Assessment of Caudate Atrophy in Huntington’s Disease

**DOI:** 10.3389/fneur.2021.616272

**Published:** 2021-04-14

**Authors:** Nina M. Mansoor, Tishok Vanniyasingam, Ian Malone, Nicola Z. Hobbs, Elin Rees, Alexandra Durr, Raymund A. C. Roos, Bernhard Landwehrmeyer, Sarah J. Tabrizi, Eileanoir B. Johnson, Rachael I. Scahill

**Affiliations:** ^1^Department of Neurodegenerative Disease, Huntington’s Disease Centre, University College London Queen Square Institute of Neurology, University College London, London, United Kingdom; ^2^Department of Neurodegenerative Disease, Dementia Research Centre, University College London Queen Square Institute of Neurology, University College London, London, United Kingdom; ^3^IXICO plc, Griffin Court, Long Lane, London, United Kingdom; ^4^Sorbonne Université, Institut du Cerveau/Paris Brain Institute AP-HP, INSERM, CNRS, University Hospital Pitié-Salpêtrière, Paris, France; ^5^Department of Neurology, Leiden University Medical Centre, Leiden, Netherlands; ^6^Department of Neurology, Ulm University, Ulm, Germany

**Keywords:** caudate, automated segmentation, Huntington (disease), FreeSurfer, FIRST, STEPS, MALP-EM

## Abstract

**Background:** Neuroimaging shows considerable promise in generating sensitive and objective outcome measures for therapeutic trials across a range of neurodegenerative conditions. For volumetric measures the current gold standard is manual delineation, which is unfeasible for samples sizes required for large clinical trials.

**Methods:** Using a cohort of early Huntington’s disease (HD) patients (*n* = 46) and controls (*n* = 35), we compared the performance of four automated segmentation tools (FIRST, FreeSurfer, STEPS, MALP-EM) with manual delineation for generating cross-sectional caudate volume, a region known to be vulnerable in HD. We then examined the effect of each of these baseline regions on the ability to detect change over 15 months using the established longitudinal Caudate Boundary Shift Integral (cBSI) method, an automated longitudinal pipeline requiring a baseline caudate region as an input.

**Results:** All tools, except Freesurfer, generated significantly smaller caudate volumes than the manually derived regions. Jaccard indices showed poorer levels of overlap between each automated segmentation and manual delineation in the HD patients compared with controls. Nevertheless, each method was able to demonstrate significant group differences in volume (*p* < 0.001). STEPS performed best qualitatively as well as quantitively in the baseline analysis. Caudate atrophy measures generated by the cBSI using automated baseline regions were largely consistent with those derived from a manually segmented baseline, with STEPS providing the most robust cBSI values across both control and HD groups.

**Conclusions:** Atrophy measures from the cBSI were relatively robust to differences in baseline segmentation technique, suggesting that fully automated pipelines could be used to generate outcome measures for clinical trials.

## Introduction

Huntington’s disease (HD) is a devastating autosomal dominant neurodegenerative disorder caused by an expansion of CAG (cytosine-adenine-guanine) trinucleotide repeat in the *huntingtin gene* (*HTT*, located on chromosome 4p16.3), encoding for huntingtin protein (1). HD patients typically have CAG repeats of >39, leading to full disease penetrance and a triad of symptoms including motor impairment, cognitive decline and psychiatric features. The mutated huntingtin protein has toxic properties causing neuronal dysfunction and death, with the medium spiny neurons in the striatum being particularly sensitive to this pathology (2). The hallmark of HD neuropathology is neurodegeneration in the caudate and putamen which becomes widespread as the disease progresses and has been demonstrated in numerous post-mortem studies (3).

Clinical trials in HD are focussed on designing and delivering disease-modifying drugs targeting mutated *HTT* RNA and DNA early in the disease or even prior to symptom onset (pre-HD) (4–8). Structural MRI has the ability to detect significant caudate atrophy and other atrophic changes in gene positive participants *in vivo* even a decade prior to symptom onset (9–12), and thus currently appears to be a promising outcome measure for clinical trials to assess efficacy of new drug therapies, when functional measures may lack sensitivity (13–16).

Currently, manual delineation is the gold standard for caudate segmentation on T1-weighted MRI imaging and has been performed in large HD observational studies (9, 10) to examine volume loss with disease progression. Manually delineating the caudate or region of interest (ROI) can achieve highly accurate volumetric outputs and considered close to “ground truth.” However, this process is labor-intensive requiring anatomical expertise, training and *a priori* information about the structure of interest, thus limiting its feasibility in large longitudinal studies with imaging data at multiple time points and in clinical trials. There are also challenges surrounding inter- and intra-rater reliability, including rater drift. There is clearly a need for alternative automated segmentation tools to aid or, potentially substitute, manual delineation in subcortical segmentation. Automated tools offer the advantage of being less time consuming, with the addition of being more flexible allowing multiple subcortical structures to be segmented simultaneously. However, the reliability and accuracy of new tools need to be examined in clinical cohorts, as many of these tools have been developed with healthy controls/atlases and not designed for highly variable brains structures including atrophied brains. Additionally, their application to imaging data across scanning platforms need to be validated as data may be affected by image quality differences (contrast, signal-to-noise ratio etc.).

Several studies have previously used automated segmentation procedures to assess caudate atrophy in both gene-positive participants not yet manifesting symptoms (pre-HD) and symptomatic HD cohorts cross-sectionally (17, 18) and longitudinally (11, 12, 19–21); few studies have cross-sectionally compared different automated tools with manual delineation of the caudate (22–25), but none have assessed the impact of baseline segmentation algorithm on longitudinal measures of change.

The aim of the current study was therefore to apply four different automated tools to the same imaging data from a cohort of controls and early HD participants and compare this with manually delineated caudates at baseline. Additionally, we investigated the Boundary Shift Integral (BSI) (26) a technique previously utilized in HD to measure longitudinal volumetric change in the caudate (cBSI) (27). The cBSI is a semi-automated technique, requiring a baseline caudate region per subject as an input. Currently this baseline region is generated by manual delineation. We compared the cBSI measure of change generated by manual caudate region with that produced by our four automated tools to determine how variability in baseline segmentation impacts this measure of change. This could potentially inform future use of automated segmentation tools and application in longitudinal datasets assessing caudate atrophy.

## Materials and Methods

### Participants

Participants were recruited from four sites (London, Leiden, Paris and Ulm) of the observational 3T MRI neuroimaging Work Package 2 (WP2) PADDINGTON study (Pharmacodynamic Approaches to Demonstration of Disease-modification in Huntington’s Disease by SEN0014196). Participants attended MRI scanning and clinical visits which included motor, cognitive and neuropsychiatric testing at baseline, 6 and 15 months from 2011 to 2013. Inclusion criteria have been published previously (28). Briefly, early HD participants (with CAG ≥ 39) were within stage 1 of the disease as defined by UHDRS Total Functional Capacity (TFC) ≥11. Disease burden (a measure of disease progression) can be calculated from age and CAG repeat length (29). Controls were spouses, partners or gene negative siblings. Participants were between 18 and 65 years of age, free from major psychiatric and concomitant neurological disorders and not participating in a clinical drug trial and able to tolerate and safely undergo MRI. The study was approved by the local ethics committees and written informed consent was obtained from each participant.

For the current study a subset of the 3T MRI baseline imaging data from 35 controls and 46 early HD participants were included for analysis retrospectively as they had passed initial quality control (i.e., no gross MRI artifacts) and were processed using automated caudate segmentation tools. Imaging data for the same participants were obtained at 15 months post baseline for the longitudinal analysis.

### Data Acquisition

3T MRI data was acquired based on protocols standardized for multi-site use (10). High-resolution three-dimensional T1-weighted structural scans were acquired on a Phillips Achieva (Leiden) and three Siemens scanners: Tim trio (London), Verio (Paris), Allegra (Ulm). In brief, for T1-weighted scans three- dimensional magnetization-prepared rapid gradient echo (MP-RAGE) protocols were used to acquire contiguous sagittal slices with 1 mm (London, Paris, Leiden) and 1.1 mm (Ulm) slice thickness, with no inter-slice gap, giving full brain coverage. The Philips parameters were TR= 7.7 ms, TE=3.5 ms, FOV= 24 cm, matrix size =224 × 224,164. For Siemens scanners TR=229 ms, TE 2.2 ms (2.81 for Ulm), FOV=28 cm, Matrix=256 × 256, 208. Quality checks of imaging data was undertaken by UCL Institute of Neurology, London (UK), to ensure compliance with relevant acquisition protocols, minimal artifacts (e.g., movement) and sufficient tissue contrast for analysis. Rescans were undertaken where necessary.

### Data Analysis

T1-weighted baseline scans were processed using five different segmentation methods using standardized procedures:

- Manual delineation as part of the PADDINGTON standard operating procedure (SOP) using in-house MIDAS (Medical information Display and Analysis System) software, as described previously (26, 27).
- FSL FIRST version 5.0.9 (FMRIB Integrated Registration and Segmentation Tool, available as part of FSL software) (30), with FSL BET performed prior to FIRST
- FreeSurfer version 5.3.0 (31).
- STEPS (Similarity and Truth Estimation for Propagated Segmentations), based on the STAPLE algorithm (32).
- MALP-EM version 1.2 (Multi Atlas Label Propagation with Expectation Maximization-Based Refinement) (33).

The baseline regions from each of these five segmentation methods were also used to produce estimates of the caudate BSI (cBSI). This uses the change in voxel intensity between two MRI scans from the same participant to infer volume change in boundaries of a defined region of interest (ROI); following baseline caudate segmentation, the ROI is expanded by two voxels which is then used to register the baseline imaging data to the repeat scan by rigid alignment. This ROI encompasses the shift of the caudate boundary between the two imaging time points. We generated the caudate atrophy value by using the three-dimensional integral of the boundary shift (in terms of voxel intensities) within this ROI as previously described (27). Different cBSI values were generated due to differences in defined caudate regions as segmented by the five different segmentation tools. Positive cBSI values represent inward boundary shift and hence tissue loss. Negative cBSI values represent outward boundary shift and thus tissue gain, likely to result from shifting tissue in response to local tissue atrophy. Image processing was performed blinded to disease status.

### Quality Control

Visual assessment and quality control (QC) of baseline caudate segmentations from the four automated tools was performed by overlaying ROIs on their original T1-weighted images and viewing them simultaneously in MIDAS. This enabled comparison of automated tools, the boundaries of segmented regions and the visible caudate boundaries on the T1 scans. Quality control was carried out blinded to disease status and study site. Scans were visually assessed in axial and sagittal planes, slice by slice, to characterize and qualitatively establish segmentation patterns, common minor errors and gross segmentation failures for each tool.

Quality control was repeated in a similar fashion for the cBSI overlays. Biologically implausible segmentations or gross failures were considered on visual assessment if segmentations did not include regions of caudate as defined by known anatomical boundaries (by the lateral ventricle medially, and the internal capsule (IC) laterally in the axial plane). Scans with minor errors, including irregular segmentation borders or outlines, lone voxels and/or visible under-/overestimations of caudate, but where the majority of segmented region was still within caudate boundaries, was classified as “pass” and included in the baseline and longitudinal analysis. In the current study no adjustments were made to any of the automated processing stages, nor was there any manual intervention or editing of the ROI following completed automated segmentation; this was to assess the default capability and reliability of the automated tools and to avoid any bias inadvertently introduced with manual intervention.

### Statistical Analysis

Quantitative analysis was carried out using STATA version 15 and IBM SPSS. For the baseline imaging data, segmented caudate volumes (in mm^3^) were extracted. The cBSI was used to assess volume change over time and normalized cBSI values were generated by dividing the cBSI values by the participants’ own baseline caudate volume to account for between participant variability in baseline volumes. Summary statistics including means, ranges, standard deviations and boxplots were produced for each method. For group comparisons of volumetrics, cBSI and normalized cBSI, independent *t*-tests and effect sizes (Cohen’s d) were calculated. To compare automated with manually derived values, paired *t*-tests and Wilcoxon signed rank test were used. Pearson’s correlation coefficients, Pitman’s test of variance and Bland-Altman plots were generated to evaluate correlation, agreement and scatter ranges. For the Bland-Altman plots, the limits of agreement were the mean difference ± 1.96 × standard deviation (SD) of difference (34). To determine the potential effects of study site on volume outputs, a generalized linear model was generated with site, disease status and interaction of these effects as predictors for the dependent variable i.e., the volume measures obtained from each method. *Post-hoc* pairwise comparisons test of estimated marginal means using Bonferroni method was performed for statistically significant results.

Similarity and accuracy between baseline manual and automated segmented ROIs were also assessed using Jaccard Similarity Coefficient/Index (35) given as a ratio, where a value close to 1 implies greater spatial overlap and better accuracy between two methods. This was calculated as below (Function 1) using the manual ROI (A) and automated ROI (B), after transformation into MNI space, to generate intersection (*A*∩*B*) and union (*A* ∪ *B*) values.

Further independent *t*-test, Mann-Whitney U test and effect sizes (Cohen’s d) were employed to assess the differences in overlap measurements in controls and HD participants for each tool. The level of significance for the statistical analysis was set to *p* < 0.05, and results are reported with 95% CI.

*Function 1: The Jaccard Index was calculated by dividing the intersection by union size*.

$$\begin{array}{l} J\;\left(A,B\right)=\frac{\left|A\;\cap \;B\right|}{\left|A\;\cup \;B\right|}=\frac{\left|A\;\cap \;B\right|}{\left|A|\;+\;|B|-|A\;\cap \;B\right|} \end{array}$$

## Results

### Demographics

Participant demographics are outlined in Table 1. Groups were well balanced and there were no significant differences in age between the early HD and the control group (t=0.9170, p=0.3621).

**Table 1:** Participant demographics.

	**Controls**	**Early HD subjects**
Number	35	46
Age mean ± SD (range)	51.03 ± 8.8 (28–66)	49 ± 9.6 (26–67)
Gender: M/F	15/20 (−1)	16 (−2)/30
Site: Leiden/London/Paris/Ulm	9/10/8 (−1)/8	12/14/8/12 (−2)
CAG repeat mean ± SD, (range)	NA	43.3 ± 2.79 (39–54)
Disease burden mean ± SD (range)	NA	371.59 ± 89.04 (226–559)

*CAG, cytosine-adenine-guanine; HD, Huntington’s disease; NA, Not applicable*.

*One female control subject from Paris and 2 male HD subjects from Ulm were excluded after quality control of segmentations (gross failure was identified, and segmentations deemed biologically implausible). These were not included in the quantitative volumetric analysis. Final sample was therefore n = 78*.

### Qualitative Assessment

Based on visual assessment of baseline segmented caudate overlaid on T1-weighted images, tools performed differently; the greatest differences were seen around the medial (between caudate and CSF/ventricle) and lateral (between caudate and IC) boundaries in the axial plane. Typical variation in tool performance with manually delineated caudate reference scan can be found in Supplementary Figure 1.

Software performance and segmentation patterns are described in Supplementary Table 1 and visual assessment with over-and underestimations of caudate volume and cBSI overlays are shown in Supplementary Figures 2–4. STEPS performed best visually, with the appearance of segmented regions looking the most similar to the manually segmented reference regions. MALP-EM was the worst-performing due to frequent underestimations of caudate boundaries on the T1-weighted image. FIRST and FreeSurfer appeared similar; FreeSurfer frequently had crude “boxed” borders, performed less well around curvatures and included non-caudate voxels. It was also noted that scans acquired at the Ulm site had more outliers, and qualitatively scans from this site appeared more noisy, although SNR was not quantified. Similar observations were made on the overlaid cBSI data, with the additional observation that MALP-EM, which often visually underestimated caudate volume, also displayed a reduced cBSI.

Of the 81 participants whose data underwent QC of the automated segmentations, three baseline scans had gross failures (Supplementary Figure 5); this included two HD participants from the Ulm site segmented by FIRST, and one control participant from Paris segmented by MALP-EM. Volume outputs from these segmentations were unreliable as they did not represent the underlying caudate structure visualized on T1-weighted image. The final number of participants for the baseline volumetric and longitudinal analysis was therefore 34 controls and 44 HD participants.

## Cross-Sectional Quantitative Analysis of the Baseline Imaging Data

### Raw Caudate Volumetrics

**Figure 1:**
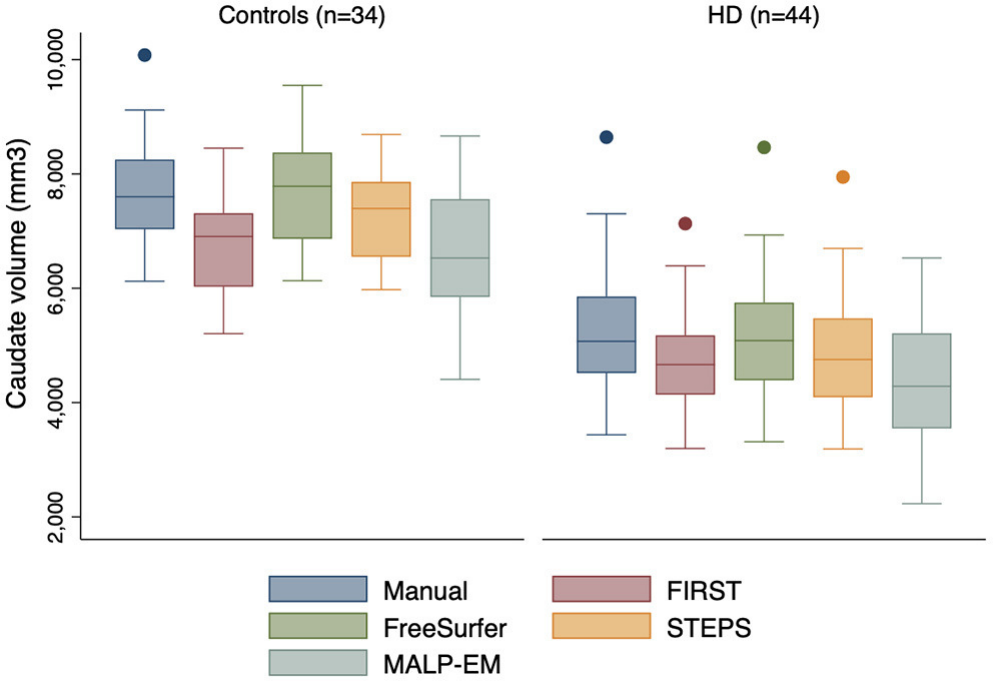
Boxplots showing caudate volumes separated by group for each segmentation tool including manual segmentation. Boxes show first quartile, median and third quartile with whiskers representing the smallest and largest volumes. Dots represent outliers. Independent *t*-tests for each method demonstrated significant volume differences at 95% CI between the two groups, all *p* < 0.0001.

Mean caudate volumes were extracted for controls and early HD patients for each method (Table 2). For all methods the mean caudate volumes were significantly higher in controls compared to early HD participants, *p* < 0.001 (Figure 1). All methods also found an association between smaller caudate volumes and greater disease burden in HD participants *p* < 0.05.

**Table 2:** Raw caudate volume and effect sizes (Cohen’s d) for all techniques (manual and automated tools).

**Method**	**Raw volume mean** **±** **SD mm**^**3**^ **(range)**		**Mean diff. mm^**3**^ (Control-HD) (95% CI)**	***p*-value**	**Cohen’s d (95% CI)**
	**Controls (*n* = 34)**	**HD (*n* = 44)**			
Manual	7674 ± 842 (6123–10080)	5168 ± 1025 (3436–8643)	2506 (2074–2938)	<0.001	2.639 (2.021–3.247)
FIRST	6779 ± 782 (5205–8451)	4715 ± 794 (3197–7130)	2064 (1706–2423)	<0.001	2.616 (2.001–3.222)
FreeSurfer	7618 ± 933 (6131–9549)	5137 ± 1024 (3314–8464)	2481 (2033–2929)	<0.001	2.517 (1.913–3.112)
STEPS	7306 ± 821 (5975–8689)	4826 ± 1007 (3189–7946)	2480 (2057–2904)	<0.001	2.664 (2.044–3.275)
MALP-EM	6666 ± 1075 (4406–8664)	4374 ± 1111 (2232–6530)	2291 (1793–2790)	<0.001	2.091 (1.530–2.644)

*Mean diff, mean difference; CI, confidence Interval; SD, standard deviation; dof, degrees of freedom*.

### Comparison of Volumetric Outputs by Software

The difference between the mean manual and automated caudate volumes were significant (*p* < 0.05) using FIRST, STEPS and MALP-EM in both HD participants and controls. While these automated tools underestimated caudate volume, no statistically significant difference in caudate volume outputs between manual and FreeSurfer were found in either HD participants (p=0.619) or controls (p=0.536) (Supplementary Table 2). A strong positive correlation between manual and automated volume outputs (with the exception of MALP-EM in controls, with only a moderate correlation, r=0.376) was demonstrated. Higher Pearson correlation coefficients between manual and automated volumes were found in HD participants compared to controls. A bias for greater volume underestimation in larger caudates was detected for FIRST (HD group only) on Pitman’s variance ratio test and illustrated on Bland-Altman plots; there was no evidence of bias for the other automated tools (Supplementary Figure 6).

### Effect of Site

**Figure 2:**
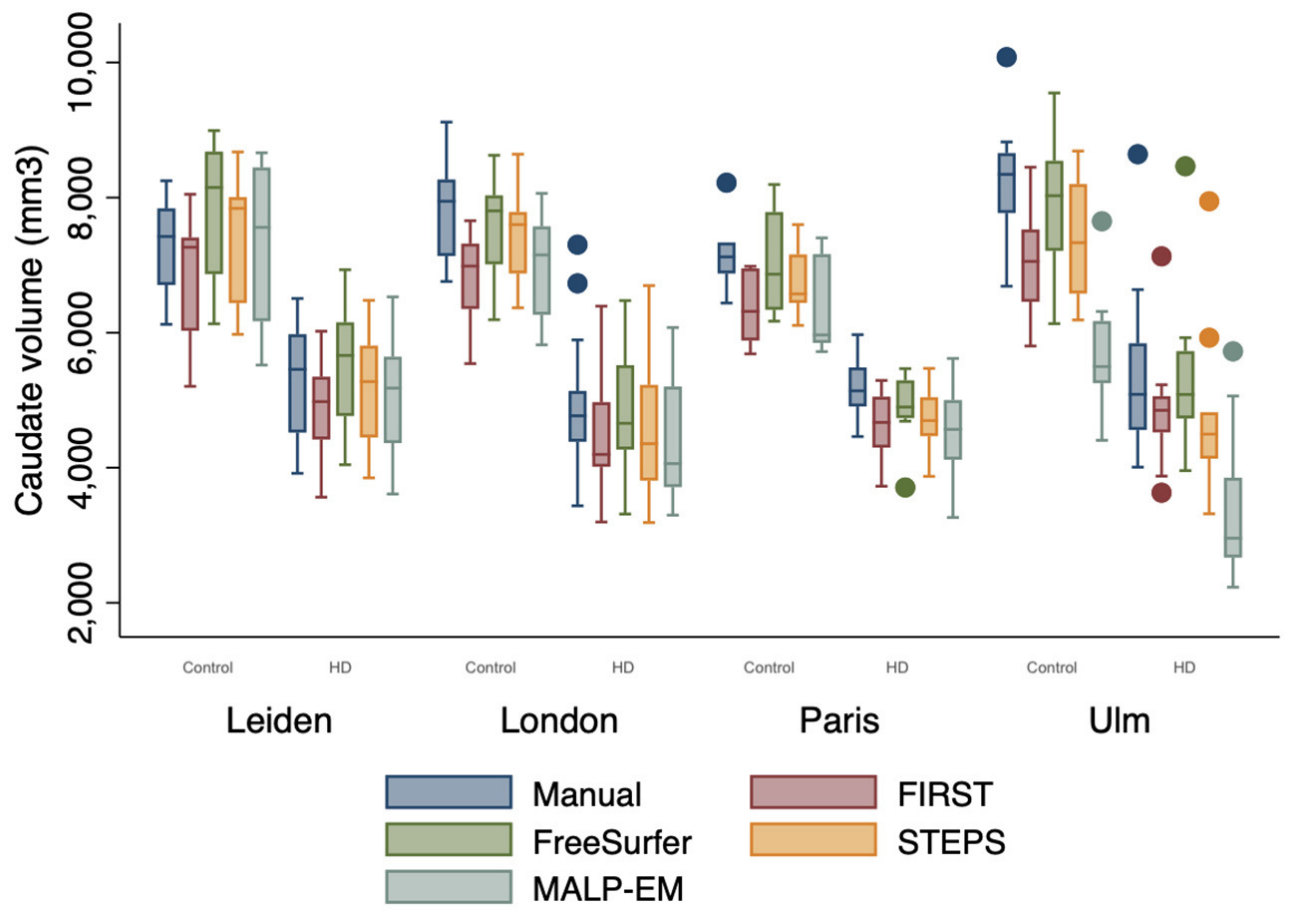
Boxplot demonstrating caudate volume outputs by disease state (HD=1, Controls=0) and site. Boxes show first quartile, median and third quartile with whiskers represent the smallest and largest volumes. Dots represent outliers. More outliers were found at the Ulm site.

Volume outputs from scans performed at the Ulm site appeared to have more outliers compared to the other sites (Figure 2). There was no statistically significant effect of site on manual, FIRST and STEPS volume measures (Supplementary Table 3). Whilst site was found to have a statistically significant effect on FreeSurfer volume measures (p=0.033), the post hoc pairwise comparisons test was non-significant. Site was also a statistically significant main effect on MALP-EM volumes, with the post hoc pairwise comparison test demonstrating significant differences between volume measures obtained at the Ulm study site and the other three sites (all *p* < 0.05) (Supplementary Table 4). The interaction between disease state and site was not a statistically significant effect on volume measures.

### Similarity Measures

**Figure 3:**
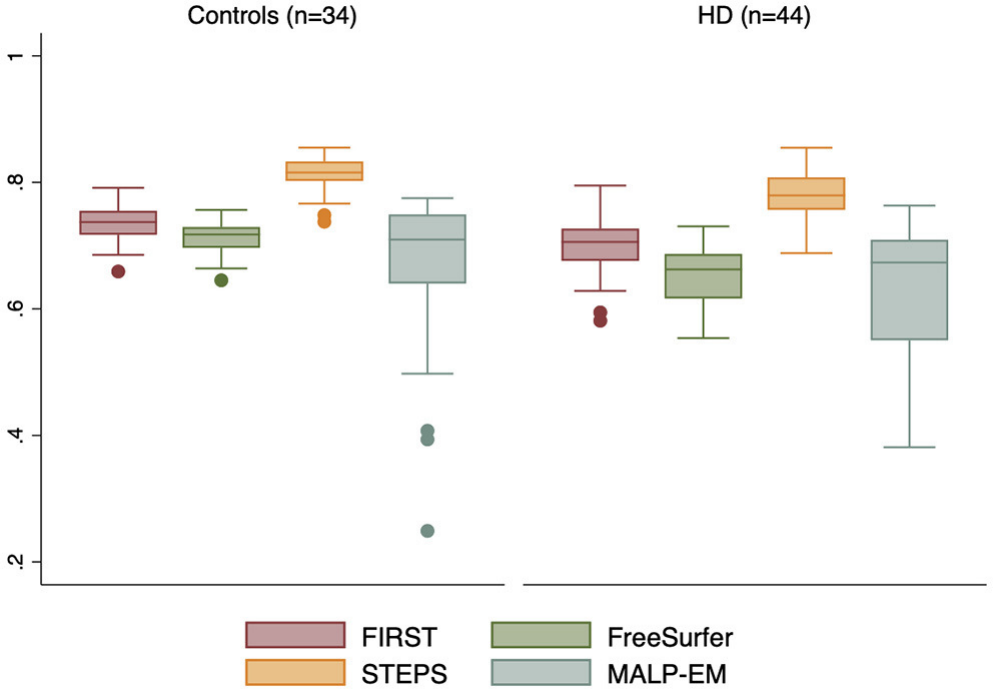
Boxplot demonstrating Jaccard Indices (as a ratio) for each automated method ROI with manual ROI. Boxes show first quartile, median and third quartile with whiskers represent the smallest and largest volumes. Dots represent outliers. STEPS segmented ROIs had larger overlaps with manually segmented ROI in both controls and HD subjects (closest to 1).

The Jaccard Indices indicated variable degree of similarity or overlap between manually segmented ROIs with each of the automated segmentations (Supplementary Table 5). Automated segmentations had greater degree of overlaps with manual ROIs in the control group compared to in HD participants, all *p* < 0.05. Effect sizes show that the largest overlap differences between controls and HD participants were with FreeSurfer segmented regions. STEPS achieved the highest level of overlap with manually segmented ROIs in both controls (0.813 ± 0.028) and HD participants (0.778 ± 0.038). STEPS also reached the highest level of overlap with a Jaccard Index of 0.855 (in both controls and HD participants), followed by FIRST, FreeSurfer and MALP-EM (Figure 3).

## Longitudinal Analysis

### cBSI and Normalized cBSI

The cBSI and normalized cBSI were significantly greater in HD participants compared to controls using all tools (all *p* < 0.05) (Table 3), demonstrating greater atrophy in HD participants over the 15-month interval. In the control group, all automated cBSI and normalized cBSI values did not significantly differ when compared to manual delineation with the exception of FIRST which reported significantly greater normalized cBSI values than those obtained with manual delineation (p=0.016). In the HD participants, FreeSurfer generated significantly smaller cBSI values (p=0.021) and normalized cBSI values (p=0.036) compared to manual delineation. There was no statistically significant difference between the MALP-EM cBSI values and those derived manually (p=0.169), however the difference became significant when cBSI was normalized to volume (p=0.001). STEPS demonstrated no significant differences in cBSI and normalized cBSI when compared to manual method in either controls or HD participants. Higher Pearson correlation coefficients were found in control participants (Supplementary Table 6). No significant bias was detected on Bland-Altman plots (Supplementary Figure 7).

**Table 3:** Group comparisons of cBSI and normalized cBSI outputs by baseline caudate segmentation method.

	**cBSI (SD), mm**^**3**^				**Normalized cBSI (SD)**			
**Baseline caudate segmentation method**	**Controls**	**HD**	**Mean diff. (control-HD) mm^**3**^ (95% CI)**	**Cohen’s d (95% CI)**	**Controls**	**HD**	**Mean diff. (control-HD) mm^**3**^ (95% CI)**	**Cohen’s d (95% CI)**
Manual	76.819 (112.470)	193.140 (131.804)	−116.320 (−172.613 to −60.028) *p* < 0.001	−0.949 (−1.409 to −0.465)	0.009 (0.014)	0.040 (0.027)	−0.031 (−0.042 to −0.020) *p* < 0.001	−1.340 (−1.832 to −0.841)
FIRST	79.840 (114.564)	194.695 (121.447)	−114.855 (−168.745 to −60.960) *p* < 0.001	−0.961 (−1.440 to −0.493)	0.011 (0.016)	0.043 (0.028)	−0.032 (−0.043 to −0.021) *p* < 0.001	−1.343 (−1.835 to −0.844)
FreeSurfer	73.518 (139.581)	172.295 (135.279)	−98.776 (−161.155 to −36.340) p=0.002	−0.720 (−1.180 to −0.256)	0.009 (0.017)	0.035 (0.029)	−0.026 (−0.037 to −0.015) *p* < 0.001	−1.049 (−1.524 to −0.569)
STEPS	78.442 (106.883)	201.298 (124.537)	−122.856 (−176.156 to −69.557) *p* < 0.001	−1.048 (−1.523 to −0.568)	0.010 (0.014)	0.045 (0.030)	−0.034 (−0.045 to −0.023) *p* < 0.001	−1.396 (−1.892 to −0.893)
MALP-EM	64.936 (119.037)	208.824 (128.769)	−143.888 (−200.570 to −87.206) *p* < 0.001	−1.154 (−1.635 to −0668)	0.010 (0.017)	0.052 (0.034)	−0.043 (−0.056 to −0.030) *p* < 0.001	−1.522 (−2.026 to −0.999)

*cBSI, caudate Boundary Shift Integral (a measure of longitudinal caudate atrophy); Mean diff, mean difference; CI, confidence Interval; SD, standard deviation*.

## Discussion

In this retrospective method comparison study, we assessed four automated segmentation tools with manual delineated caudate in early HD participants and controls across four sites. The aim was to identify tools that could reliably segment caudate regions (using default settings without manual intervention) and potentially substitute manual delineation in future clinical trials with large imaging datasets to track disease progression. We characterized the segmentation outputs qualitatively and quantitatively and examined volume outputs, overlap measures, cBSI and normalized cBSI to establish which tools produced the most similar results to that of manual delineation. Although we have demonstrated that automated segmentation techniques can be used as reliable alternatives to manual delineation, performance can vary depending on the specific tool used, factors related to study site and disease status.

The study highlights the importance of visual QC of segmented imaging data. We found that FreeSurfer demonstrated similar qualitative performance to that previously reported in a much smaller pre-HD cohort (23). Whilst MALP-EM has demonstrated good visual accuracy for cortical gray matter segmentation in HD patients (36), MALP-EM and STEPS are relatively unexplored tools in subcortical segmentation. To our knowledge we are the first to report on caudate segmentation patterns and errors by these tools in a pathological cohort and compare this to manual delineation. While not all users of automated tools undertake QC of segmentations, we detected three failed segmentations which were excluded from the quantitative analysis. Although this was a small number of exclusions, in rare neurodegenerative disorders like HD or in studies with small sample sizes, this could still be significant; the effect of visual QC using FreeSurfer in cortical segmentations have shown to improve reliability and accuracy of segmented imaging data, leading to a substantial decrease in the number of participants needed to be enrolled in a study to detect significant group differences or compensate for increased variance introduced by imaging data not having undergone or passed visual QC (37).

Additionally, on QC we observed that the imaging data obtained from the four study sites differed in quality, despite having employed similar sequence parameters; It is unclear why the T1 weighted MRI images differed in terms of contrast (between gray and white matter structures) and noise, particularly from the Ulm Site. It was felt that despite standardization of acquisition parameters as far as possible, inevitably there are differences between scanner manufacturers (Siemens and Philips) employed at the different sites. The majority of outliers were found in the Ulm HD- group and this may mirror the qualitative findings. There may also have been underlying greater variance in the caudate volumes in the HD participants at this site, as outliers were seen across all methods including manual volumes. These observations were reflected in our generalized linear model, where site had a global effect on MALP-EM volume measure outputs; significantly smaller caudate volumes were obtained from imaging data at Ulm compared to the other sites using MALP-EM. Whilst site appeared to have a global effect on FreeSurfer volume outputs, the post hoc pairwise comparison test was not significant; this may be due to a combination of a weaker significant global effect observed with FreeSurfer and lack of statistical power due to small sample size. Whilst there will be challenges with multi-site MR data in terms of across- scanner variation and image quality, with possible impact on performance of automated tools, it is felt that the inclusion of multi-center data allows for overall larger sample size (which is of particular importance in rare diseases such as HD) and increased generalisability of the results. Overall, these findings may also indicate that some tools are more data driven, and that their performance is dependent on the quality of the input imaging data, which is predominately determined by scanner used, sequence parameters etc. QC of imaging data is therefore not only essential to determine the reliability of tools to segment anatomically accurate regions for inclusion in a quantitative analysis, but also crucial in deciding what tool to employ in an imaging study given the quality of imaging data to be analyzed and suitability for the specific tool.

A comparison of baseline volumetrics demonstrated that all automated tools could establish group separation in this subset of early HD participants and controls, with HD participants having significantly smaller caudate volumes. Longitudinally, the cBSI and normalized cBSI values showed significant increased caudate atrophy rates in HD participants compared to controls. Both these findings reflect well-known disease characteristics (11, 18–20, 38). Automated tools (FIRST, STEPS and MALP-EM) generated significantly smaller caudate volumes than those derived manually which is consistent with previous reports using other tools such as BRAINS (12), but in contrast to an automated ABV (atlas based volumetry using a binary caudate mask derived from the LONI probabilistic brain atlas) tool that systematically derived larger volumes (24). Whilst FreeSurfer volumes were most similar to those derived manually, the overlap analysis was more in keeping with the qualitative findings, where higher Jaccard Indices were found between manual and STEPS segmented ROIs in both groups. Although FreeSurfer performed well in the simple volumetric comparison, the slightly poorer overlaps between FreeSurfer and manually segmented ROIs demonstrate that reporting volumetric outputs only, when attempting to validate and assess the utility of tools, is inadequate since these measures do not necessarily reflect spatial agreement between two segmented ROIs. Previous Dice similarity coefficient (DSC) reported by Khan et al. for FreeSurfer and manually segmented caudate in pre-HD was 0.77 (right) and 0.80 (left) (23). The Jaccard Index is closely related to DSC (where DSC=2J/1+J) (39), thus calculated mean FreeSurfer DSC in the current study (0.831 for controls and 0.790 in early HD) demonstrate similar levels of overlap potentially across the HD disease spectrum.

Longitudinally the cBSI and normalized cBSI values were greater in HD participants compared to controls, reflecting the expected increased caudate atrophy rates in HD subjects exceeding the effects of normal aging (18). Hobbs et al. have previously reported increased between-subject variability in caudate volume in HD compared to controls (40), and to account for this, we normalized cBSI to baseline caudate volumes. In the current study, the normalized cBSI measures also led to complete group separation across all segmentation tools, demonstrating that this may be a reliable way to calculate caudate volume change while also accounting for the between-subject variability in baseline caudate volumes. STEPS, which had underestimated baseline volumetrics, was the only tool unaffected by this normalization however, demonstrating no statistically significant difference with manually derived cBSI or the normalized cBSI measures. The findings could indicate that either the baseline volumetric inaccuracy was not large enough to impact on the subsequent cBSI calculations, or that the baseline caudate volume itself does not greatly affect the cBSI. This seems plausible considering that despite FreeSurfer producing the most similar baseline volumetric outputs to those derived manually, it generated smaller cBSI measures than manually derived measures, becoming significant in the HD group. Automated tools that are unable to generate accurate volume change measures over time would not be reliable in clinical trials assessing treatment effects.

As with the overlap analysis, where all techniques demonstrated greater overlaps in controls compared to HD participants, suggesting that automated tools may be less accurate in atrophied brains, this may also be the case for cBSI measures; from the longitudinal analysis we observed that tools, such as MALP-EM, overestimated normalized cBSI in HD participants but not controls. Some automated tools may be less accurate in generating reliable measures in atrophied brains, both at baseline and longitudinally where atrophic changes occur at an increased rate. However, this would perhaps not impact on their utility in future pre-HD studies, as this group is expected to be more “similar” to normal controls with less extreme subcortical atrophy, compared to that observed in the early HD participants in the current study, but this would need further validation. Studies would also need to assess whether tools would be sensitive to smaller atrophic changes than those observed here with participants in stage 1 of the disease and with imaging data at 15 months from baseline.

A limitation is that more tools could have been assessed, but we included two widely used tools (FIRST and FreeSurfer) along with two unexplored tools (STEPS, MALP-EM) not previously validated in subcortical caudate segmentation in HD. Although STEPS performed well in the current study, a major limitation to its use and application in other clinical cohorts is that it is not currently freely available. Greater consistency between scans that were deemed as a “pass” could also have been established; MALP-EM segmentations for instance, frequently underestimated caudate volume, but passed quality control as segmented ROIs were still considered to be within anatomical boundaries. By including these in the volumetric analysis it was not unexpected that the mean volumes appeared smaller for MALP-EM. The types of segmentation errors seen with MALP-EM (including “holes”) were not present with any of the other tools, thus excluding a number of scans with these errors would have wrongfully represented MALP-EM’s ability to accurately carry out segmentations in this cohort. This would also have impacted on the purpose of the study, which was to assess default performance of segmentation tools. Additionally, raw caudate volumes reported for each tool were not normalized for total intracranial volume (TIV). This may have impacted on group comparisons, but this was not the main focus of the study.

## Conclusion

The study has implications for future HD studies, but results can be extended to other neurodegenerative or pathological cohorts where using automated segmentation tools are becoming essential for large cohorts. Imaging markers that can be sensitively measured to distinguish pathological cohorts from controls are vital for study design development. Equally important is the employment of validated tools that can accurately and reliably detect and longitudinally track disease progression, enabling assessment of treatment effects in clinical trials. When evaluating which automated tool to use, it will be important to establish how tools have been validated; reporting only raw baseline automated volumetric output comparisons with manually derived volumetrics cannot reliably validate the accuracy of the automated tool. Similarly, the cohort of which the tool has been validated in will be important; as demonstrated in the current study, all tools had poorer overlaps with manually segmented regions in HD participants compared to controls, presumably due to the inability of tools to fully cope with abnormal brain configuration and atrophy.

Ultimately, the best tools would output similar volumes as manually delineated outputs, demonstrate close overlaps (using similarity measures such as Jaccard Index or DSC) with manually segmented ROIs and demonstrate reliable measures of volume change over time. In the current study FreeSurfer demonstrated similar volume outputs but had slightly poorer overlaps and significantly different cBSI measures in the HD group. MALP-EM segmentations were generally poor and FIRST performed less well on baseline and longitudinal measures and would therefore be considered less reliable in the assessment of caudate volume cross -sectionally and longitudinally in this cohort. STEPS was qualitatively the most accurate demonstrating the greatest overlaps, and the significantly smaller cross-sectionally derived volume outputs did not seem to impact on its ability to generate reliable cBSI measures longitudinally. This indicates that when measuring volume change over time, using a tool such as STEPS that have demonstrated better measures of similarity with manual delineation may be more appropriate, keeping in mind that absolute volumes may be systematically underestimated.

## Data Availability Statement

The data analyzed in this study is subject to the following licenses/restrictions: The datasets presented in this article are not readily available in compliance with the participant informed consent. Requests to access these datasets should be directed to r.scahill@ucl.ac.uk.

## Ethics Statement

The studies involving human participants were reviewed and approved by the Central London Research Ethics Committee 4–Reference number 11/H)715/2. The patients/participants provided their written informed consent to participate in this study.

## Author Contributions

NM contributed to study design, data collection, analyzed the data, and wrote the manuscript. TV contributed to study design, data collection, analysis, and preparation of the manuscript. IM contributed to data analysis and review of the manuscript. NH contributed to data collection, data analysis, and review of the manuscript. ER contributed to data collection and review of the manuscript. ST, AD, RR, and BL were PIs for the PADDINGTON study, with overall responsibility for all participants, and reviewed the manuscript. EJ contributed to study design, data collection, and preparation of the manuscript. RS developed the study, contributed to data collection, and preparation of the manuscript. All authors contributed to the article and approved the submitted version.

## Conflict of Interest

ER was employed by UCL during the period of data collection and image processing. She now works for IXICO plc. The remaining authors declare that the research was conducted in the absence of any commercial or financial relationships that could be construed as a potential conflict of interest.
