# Supplementary material for: Validating Automated Segmentation Tools in the Assessment of Caudate Atrophy in Huntington’s Disease
Source: Front Neurol. 2021 Apr 14;12:616272. doi: 10.3389/fneur.2021.616272 (PMC8079754; doi:10.3389/fneur.2021.616272)
Supplement: Supplementary file 1 [file Data_Sheet_1.docx]

**Supplementary Material**

*
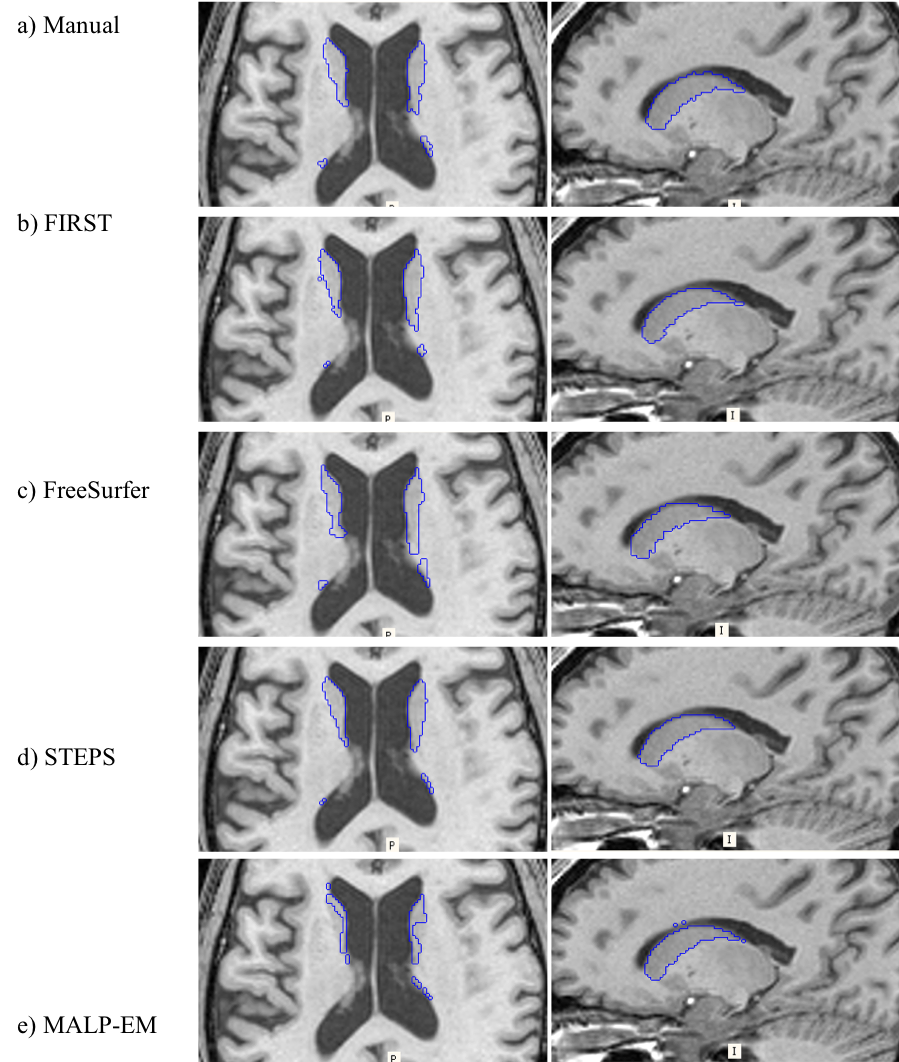
*

Supplementary Figure 1a-e: Visual assessment and comparison of caudate segmentations of a baseline scan with manual reference (a) and the four automated tools (b-e) in a typical study subject. There was significant variability in the segmentations when visualised in the axial plane (left column) using all tools when compared to the manually segmented refence ROI (top left). This is in contrast to the overall good visual accuracy of all automated tools compared to manual segmentation (top right) in the sagittal plane (right column).

*
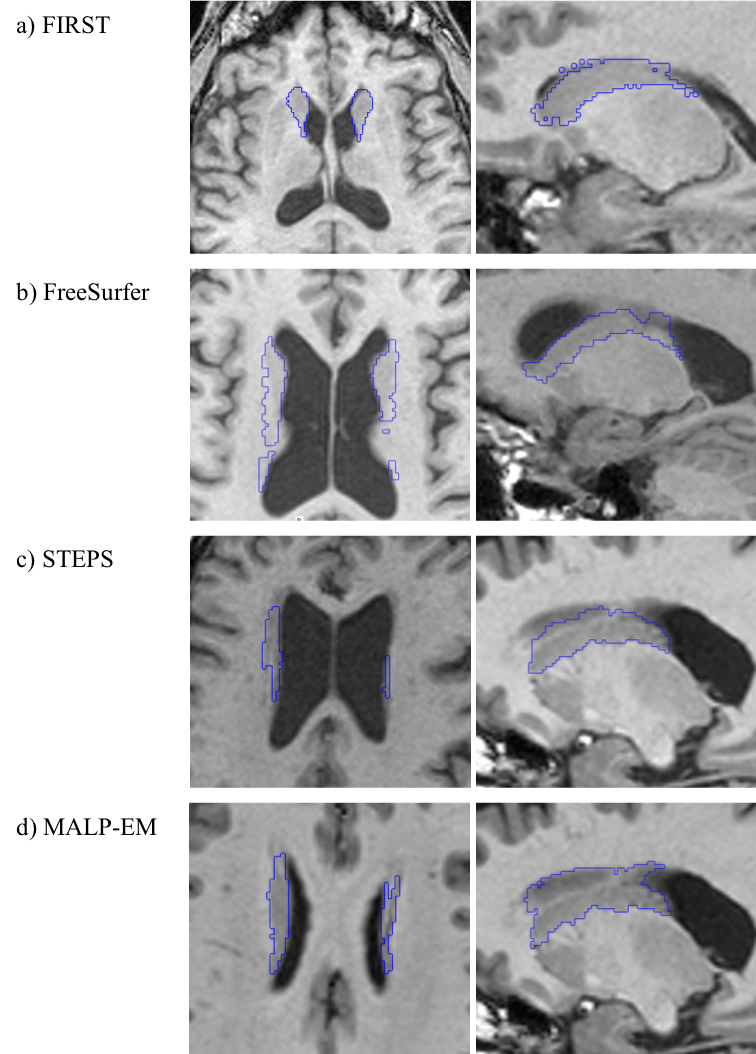
*

Supplementary Figure 2a-d: Minor errors and segmentation patterns in axial (left column) and sagittal planes (right column), where automated tools **overestimated** caudate volume: a) minor ventricular spillage (axial) and random lone voxels seen superior and anterior to the lateral ventricle (sagittal). b) Non-smooth segmentation borders with WM voxels included laterally, with additional GM voxels excluded medially (axial). Inclusion of ventricular voxels (sagittal). c) Overestimation seen laterally (axial), and inclusion of ventricular voxels (sagittal). d) Lateral overestimation bilaterally (axial), and inclusion of non-caudate voxels superiorly (sagittal).

*
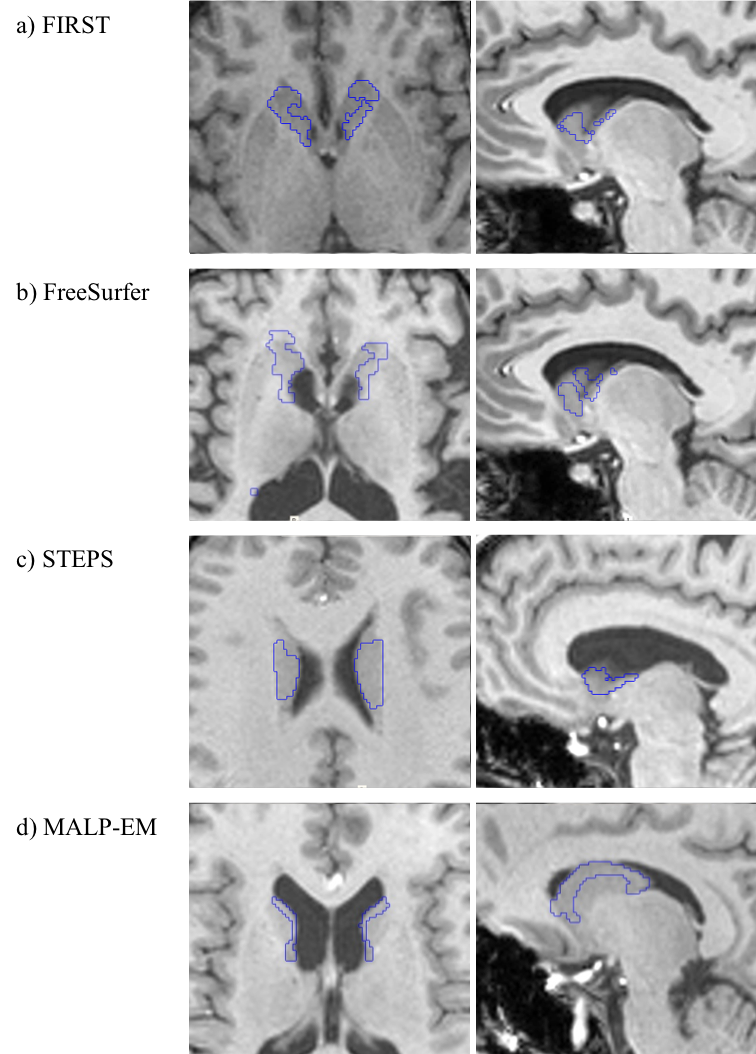
*

Supplementary Figure 3a-d: Minor errors and segmentation patterns in axial (left column) and sagittal (right column) planes where automated tools **underestimated** caudate volume: a) Medial underestimation (axial), and caudate head voxels missed (sagittal). b) Underestimations medially, but with WM voxels included anteriorly (axial) and “splitting” of segmented region (sagittal). c) “Missed” GM voxels anteriorly (axial) with underestimation due to “tight” segmentation (sagittal). d) Caudate voxels exclusions laterally bilaterally (axial) and inferiorly, with minor WM voxels inclusions anteriorly (sagittal).


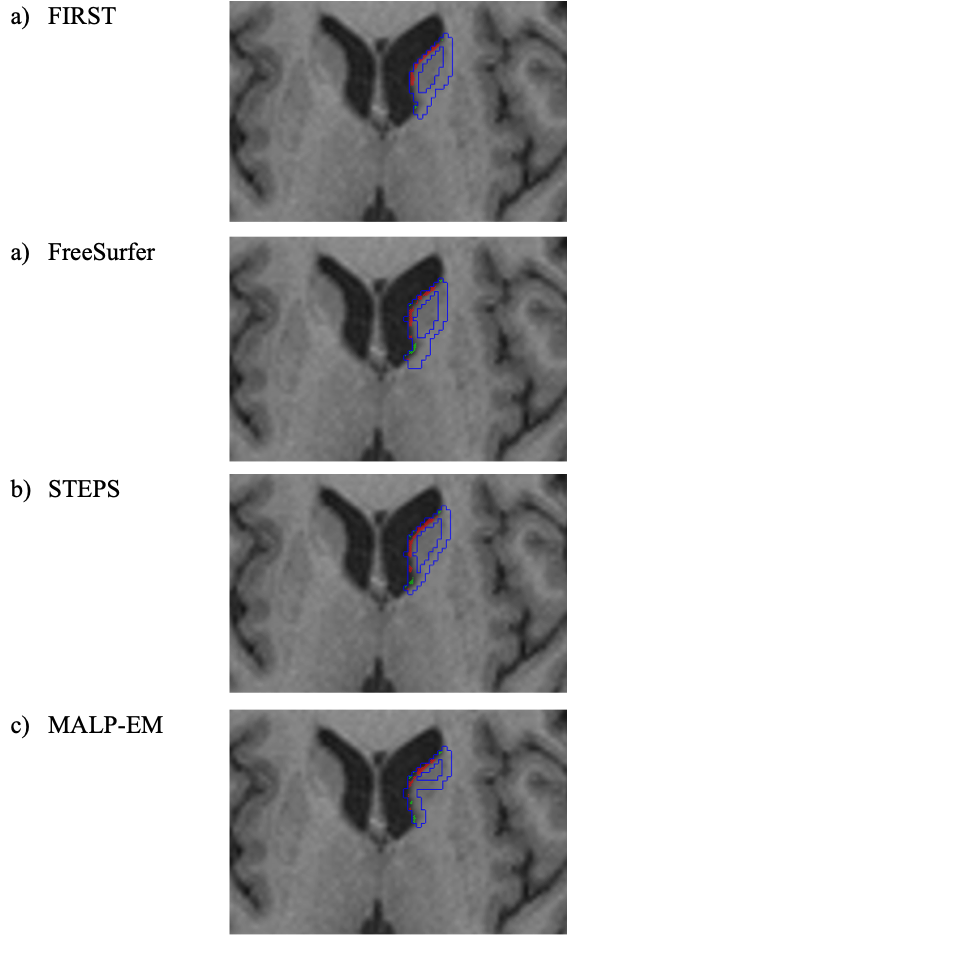


Supplementary Figure 4 a-d: Magnified examples of BSI overlays on caudate segmentation by all four methods. Blue lines indicate segmentation boundaries. Red pixels indicate BSI “loss” or caudate atrophy and green pixels indicate BSI “gain” or shifting tissue towards ventricles as a result of caudate atrophy.

*
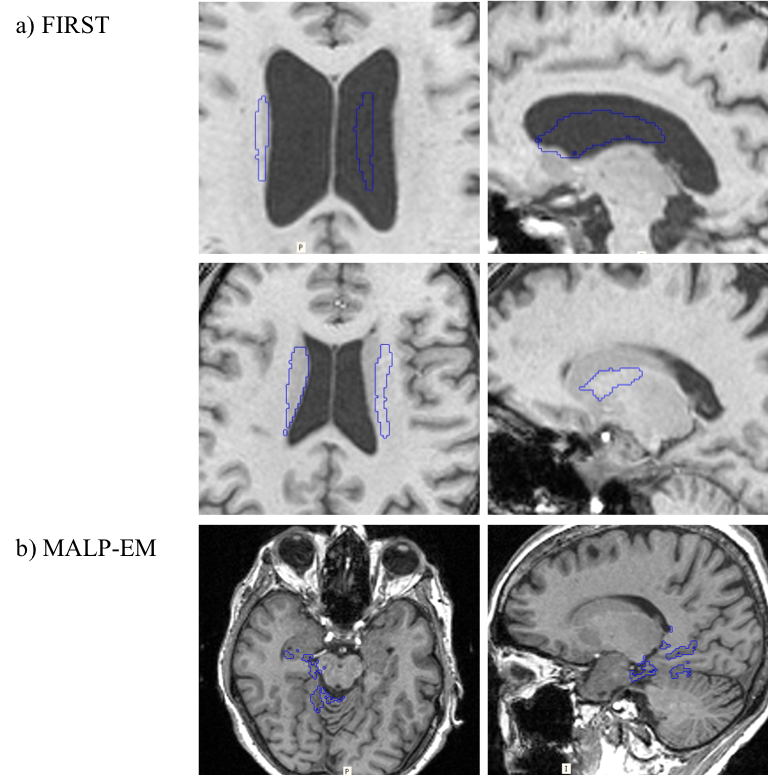
*

Supplementary Figure 5a, b: Three failed segmentations (FIRST n=2, both HD subjects in Ulm, MALP-EM n=1, control subject in Paris) detected on quality control and not included in quantitative analysis: a) FIRST segmentations missed caudate completely (unilateral) with segmentation in the lateral ventricle (top row) and in WM (middle row). b) MALP-EM segmentation in brainstem and cerebellum (bottom row).

|  | Controls (n=34) | HD (n=44) |
| --- | --- | --- |
| FIRST | 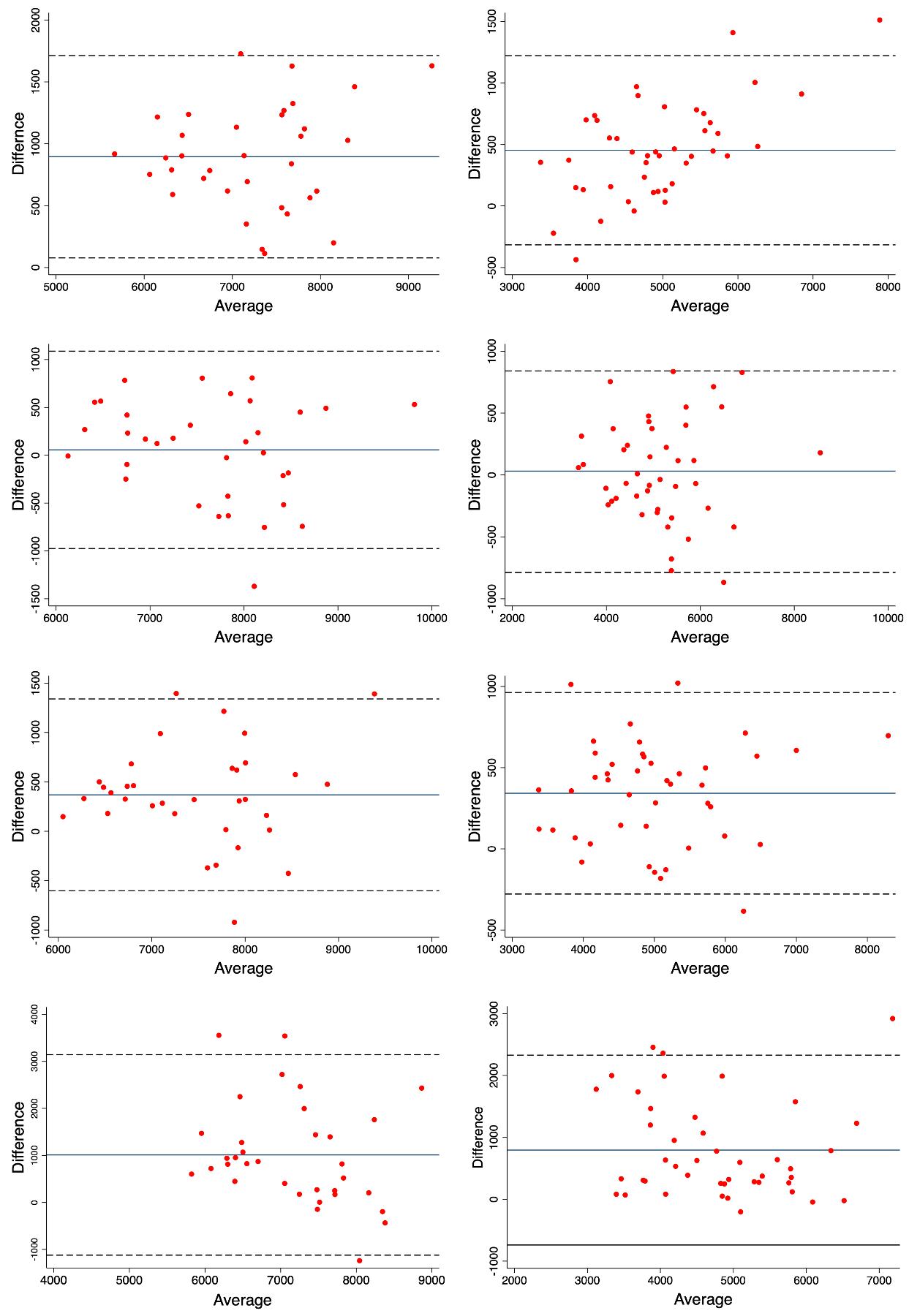 | |
| FreeSurfer |  |  |
| STEPS |  |  |
| MALP-EM |  |  |

Supplementary Figure 6: Bland- Altman plots comparing manual and automated caudate volumes (mm^3^) for each method in controls and HD subjects. Difference=Manual volume-automated volume, Average=(Manual volumes + automated volumes)/2.

|  | Controls (n=34) | HD (n=44) |
| --- | --- | --- |
| FIRST | 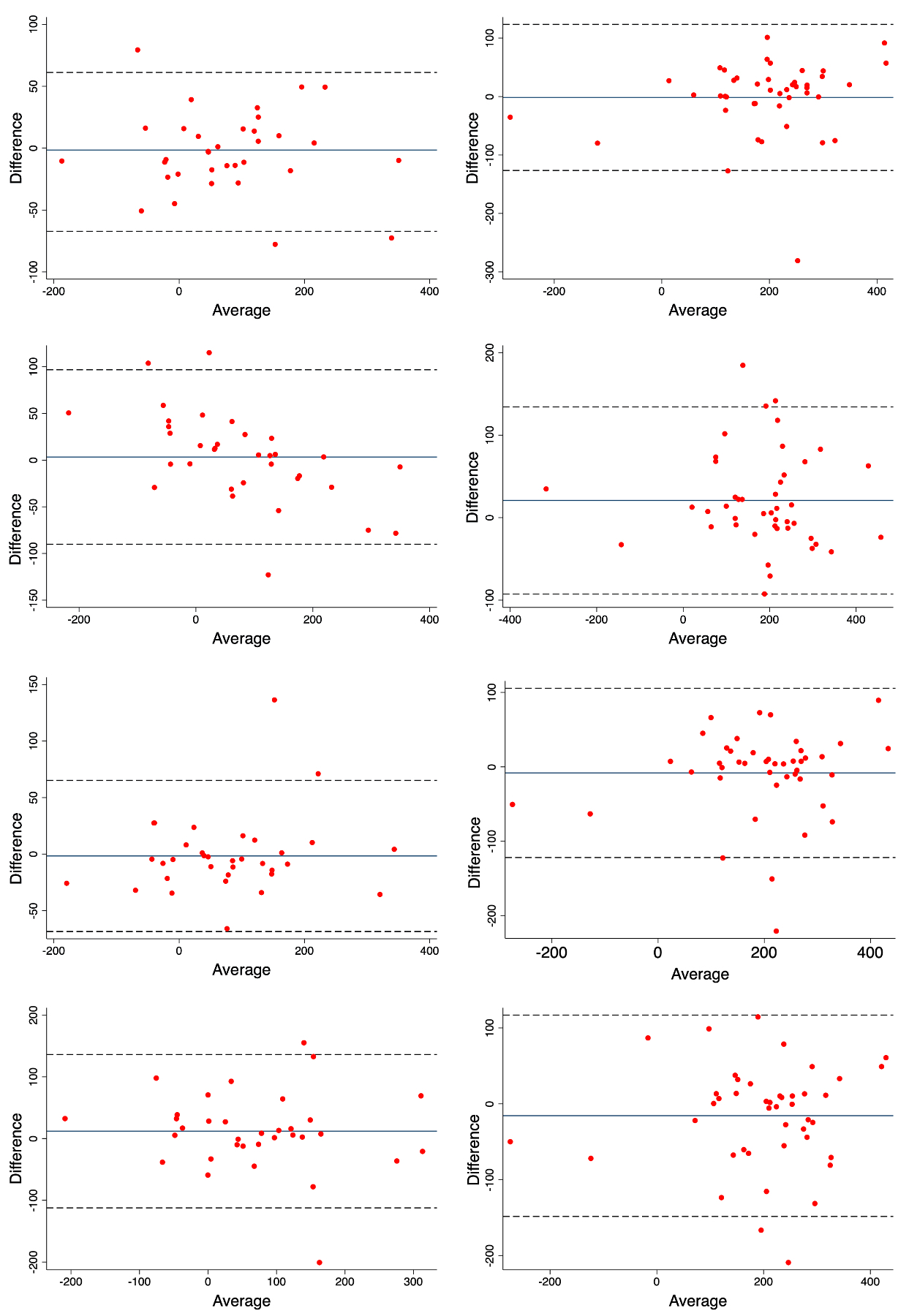 | |
| FreeSurfer |  |  |
| STEPS |  |  |
| MALP-EM |  |  |

Supplementary Figure 7: Bland- Altman plots comparing manual and automated caudate cBSI (mm^3^) for each method in controls and HD subjects. Difference=Manual cBSI-automated cBSI, Average=(Manual cBSI + automated cBSI)/2.

Supplementary Table 1. Automated tools performance with details of some common patterns and minor errors of segmentation.

| **Software** | **Software performance and segmentation patterns** |
| --- | --- |
| **FIRST** | Sagittal:   - Irregular non-smooth and jagged segmentation borders, but generally good GM/WM differentiation superiorly and inferiorly. - Sometimes appeared to be “generous” in segmentations compared to other techniques with frequent WM and ventricular spillage with inappropriate voxel inclusions. - Liable to caudate underestimation in planes where head of caudate is small.   Axial:   - Less good segmentation seen around curvatures (lateral ventricles) with loss of periventricular GM voxels. - Struggled to include appropriate GM voxels medially and anteriorly in planes with small caudate head. - Some random/lone voxels included.   2 instances of gross failure, where left caudate was completely missed by this segmentation tool. |
| **FreeSurfer** | Sagittal:   - Common error with inclusions of WM voxels superior to lateral ventricle. - Commonly included non-caudate voxels anteriorly.   Axial:   - Generally, “boxed appearance” around caudate with crude, non-smooth borders with overestimations anteriorly, laterally and centrally. - “Splitting” of segmented region despite underlying uniform caudate structure in some scans.   No segmentation fails. |
| **STEPS** | Sagittal   - Smoothest segmentation borders and appearances were most similar to manually segmented caudate. In a few cases segmentations appeared tight (compared to FIRST). - Appeared more inclusive of caudate volume even in planes where head of caudate was small (compared to other techniques).   Axial   - Some minor underestimations of caudate head in planes when this structure and/or lateral ventricles were small (appeared tight).   No segmentation fails. |
| **MALP-EM** | Sagittal   - Frequent underestimation of caudate volume inferiorly through most slices in affected scans. - Frequent overestimation with WM voxels inclusions superior to lateral ventricle.   Axial   - Usually first to segment (superiorly) compared to other techniques (slice-by-slice). - Frequent underestimation of caudate voxels laterally. - Frequent lone voxels, “holes” in segmented region despite uniform caudate structure on underlying T1-weighted image. - Poor segmentation around head of caudate.   1 instance of gross failure. |
| Abbreviations: GM=Grey matter; WM=White matter | |

Supplementary Table 2. Mean manual volume comparison with each automated volume output, Pitman´s ratio of variance and Pearson correlation coefficient, r.

| **Method** | **Group** | **Mean diff. mm^3^**  **(Manual-Automated) (95% CI)** | **Pitman’s variance ratio (ratio of SD) (95% CI)** | **Pearson, r** |
| --- | --- | --- | --- | --- |
| **FIRST** | Controls  (n=34) | 895 (749-1041)  p<0.001 | 1.076 (0.902-1.283)  p=0.450 | 0.870 |
|  | HD  (n=44) | 453 (334-573)  p<0.001 | 1.290 (1.159-1.436)  p<0.001 | 0.938 |
| **FreeSurfer** | Controls  (n=34) | 56 (-127-249)  p=0.536 | 0.902 (0.739-1.102)  p=0.304 | 0.829 |
|  | HD  (n=44) | 31 (-94-157)  p=0.619 | 1.001 (0.885-1.131)  p=0.992 | 0.919 |
| **STEPS** | Controls  (n=34) | 368 (195-541)  p<0.001 | 1.024 (0.836-1.255)  p=0.812 | 0.823 |
|  | HD  (n=44) | 342 (246-439)  p<0.001 | 1.019 (0.925-1.120)  p=0.771 | 0.952 |
| **MALP-EM** | Controls  (n=34) | 1009 (629 - 1388)  p<0.001 | 0.783 (0.564 – 1.087)  p=0.142 | 0.376 |
|  | HD  (n=44) | 794 (556 – 1032)  p<0.001* | 0.922 (0.747 -1.137)  p=0.442 | 0.734 |
| Abbreviations: Mean diff= mean difference; CI: confidence Interval, SD= standard deviation; dof= degrees of freedom.  *Wilcoxon signed rank test for MALP-EM as difference data generated did not meet assumptions for paired t-test. | | | | |

Supplementary Table 3. Generalised linear model with main effects of site, disease status and interaction of main effects on volume measures obtained for each method.

| **Dependent volume measures** | **Main model effects** | **df** | **Wald Chi-square** | **p-value** |
| --- | --- | --- | --- | --- |
| **Manual** | Disease status | 1 | 145.360 | <0.05 |
|  | Site | 3 | 5.611 | 0.132 |
|  | Interaction | 3 | 4.554 | 0.208 |
| **FIRST** | Disease status | 1 | 136.336 | <0.05 |
|  | Site | 3 | 3.849 | 0.278 |
|  | Interaction | 3 | 0.692 | 0.692 |
| **FreeSurfer** | Disease status | 1 | 134.526 | <0.05 |
|  | Site | 3 | 8.712 | 0.033* |
|  | Interaction | 3 | 1.212 | 0.750 |
| **STEPS** | Disease status | 1 | 143.318 | <0.05 |
|  | Site | 3 | 3.836 | 0.280 |
|  | Interaction | 3 | 2.403 | 0.493 |
| **MALP-EM** | Disease status | 1 | 118.532 | <0.05 |
|  | Site | 3 | 32.565 | <0.05 |
|  | Interaction | 3 | 1.330 | 0.722 |

Abbreviations: df= Degrees of freedom.

*Significant global effect on the main model which could not be detected on subsequent pairwise comparisons using estimated marginal means with Bonferroni correction.

Supplementary Table 4. Statistically significant results from post hoc pairwise comparison for MALP-EM volumes.

| **Site A** | **Site B** | **Mean diff.**  **(A-B)** | **Std.Error** | **df** | **Bonferroni Sig.** | **95% Wald CI for difference** |
| --- | --- | --- | --- | --- | --- | --- |
| Ulm | Leiden | -1627.582 | 291.826 | 1 | <0.05 | -2397.493 to - 857.670 |
|  | London | -1153.014 | 283.706 | 1 | <0.05 | -1901.504 to - 404.524 |
|  | Paris | -915.543 | 316.335 | 1 | 0.023 | -1750.113 to - 80.970 |
|  |  |  |  |  |  |  |

Abbreviations: Mean diff.=Mean difference, Std. Error =Standard error, df= Degrees of freedom, CI= Confidence interval.

Supplementary Table 5. Measure of similarity with mean Jaccard Indices for all techniques (where the Jaccard Index is calculated as manual and automated ROI intersection divided by the manual and automated ROI union).

| **Method** | **Mean Jaccard Index ± SD (range)** | | **Mean Diff. mm^3^**  **(Control-HD) (95% CI)** | **p-value** | **Cohen´s d (95% CI)** |  |
| --- | --- | --- | --- | --- | --- | --- |
|  | **Controls**  **(n=34)** | **HD subjects**  **(n=44)** |  |  |  |  |
| **FIRST** | 0.735 ± 0.030  (0.659-0.791) | 0.700 ± 0.042  (0.581-0.794) | 0.035 (0.018-0.052) | <0.001 | 0.939  (0.465-1.408) |  |
| **FreeSurfer** | 0.712 ± 0.025  (0.645-0.756) | 0.653 ± 0.046  (0.554-0.730) | 0.059 (0.041-0.076) | <0.001 | 1.534  (1.026-2.046) |  |
| **STEPS** | 0.813 ± 0.028  (0.738-0.855) | 0.778 ± 0.038  (0.688-0.855) | 0.035 (0.019-0.050) | <0.001 | 1.029  (0.549-1.502) |  |
| **MALP-EM** | 0.668 ± 0.120  (0.394-0.775) | 0.625 ± 0.118  (0.381-0.763) | 0.0426 (-0.012-0.097) | 0.034* | 0.362  (-0.091-0.812) |  |
| Abbreviations: Mean Diff= Mean difference, CI: confidence Interval, SD= standard deviation.  *Two sample Wilcoxon rank sum test for MALP-EM data as did not meet t-test assumptions. | | | | | | |

Supplementary Table 6: Comparison of cBSI and normalised cBSI generated by automated tools and manual method.

|  |  | **cBSI** | | | **Normalised cBSI** | | |
| --- | --- | --- | --- | --- | --- | --- | --- |
| **Method** | **Group** | **Mean diff. mm^3^**  **(Manual-Automated) (95% CI)** | **Pitman’s variance ratio (ratio of SD) (95% CI)** | **Pearson, r** | **Mean diff. mm^3^**  **(Manual-Automated) (95% CI)** | **Pitman’s variance ratio (ratio of SD) (95% CI)** | **Pearson, r** |
| **FIRST** | Controls  (n=34) | -3.021  (-14.444 to 8.403)  p=0.594 | 0.982 (0.886 to 1.088)  p=0.716 | 0.959 | -1.773  (-3.569 to 0.238)  p=0.016* | 0.860 (0.769 to 0.962)  p=0.010 | 0.950 |
|  | HD  (n=44) | -1.555  (-20.944 to 17.833)  p=0.283* | 1.085 (0.935 to1.260)  p=0.277 | 0.876 | -3.354  (-7.576 to -0.670)  p=0.283* | 0.967 (0.836 to 1.118)  p=0.640 | 0.883 |
| **FreeSurfer** | Controls  (n=34) | 3.300  (-13.340 to 19.941)  p=0.689 | 0.806 (0.721 to 0.901)  p<0.001 | 0.951 | 0.194  (-1.969 to 2.357)  p=0.856 | 0.792 (0.704 to 0.890)  p<0.001 | 0.945 |
|  | HD  (n=44) | 20.845  (3.257 to 38.433)  p=0.021 | 0.974 (0.855 to 1.111)  p=0.692 | 0.907 | 4.451  (0.293 to 8.609)  p=0.036 | 0.937 (0.815 to 1.078)  p=0.355 | 0.893 |
| **STEPS** | Controls  (n=34) | -1.6228  (-13.520 to 10.275)  p=0.139* | 1.053 (0.944 to 1.173)  p=0.349 | 0.953 | -0.874  (-2.411 to 0.683)  p=0.054* | 0.981 (0.876 to 1.099)  p=0.734 | 0.949 |
|  | HD  (n=44) | -8.159  (-25.798 to 9.480)  P=0.907* | 1.058 (0.924 to 1.212)  p=0.406 | 0.899 | -4.869  (-9.010 to -0.728)  p=0.102* | 0.907 (0.789 to 1.043)  p=0.167 | 0.893 |
| **MALP-EM** | Controls  (n=34) | 11.884  (-10.222 to 33.990)  p=0.169* | 0.945 (0.783 to 1.140)  p=0.544 | 0.852 | -0.055  (-3.195 to 3.084)  p=0.972 | 0.810 (0.670 to 0.980)  p=0.031 | 0.847 |
|  | HD  (n=44) | -15.684  (-36.247 to 4.889)  p=0.132 | 1.024 (0.876 to 1.196)  p=0.765 | 0.865 | -12.534  (-19.219 to -5.849)  p=0.001* | 0.801 (0.656 to 0.977)  p=0.029 | 0.766 |
| Abbreviations: Mean diff= mean difference; CI: confidence Interval, SD= standard deviation.  *Wilcoxon signed rank test used as difference data generated did not meet assumptions for paired t-test. | | | | | | | |
